# Observations on the Effects of Residualization and Dehalogenation on the Utility of *N*-Succinimidyl Ester Acylation Agents for Radioiodination of the Internalizing Antibody Trastuzumab

**DOI:** 10.3390/molecules24213907

**Published:** 2019-10-30

**Authors:** Satish K. Chitneni, Eftychia Koumarianou, Ganesan Vaidyanathan, Michael R. Zalutsky

**Affiliations:** 1 Department of Radiology, Duke University Medical Center, Durham, NC 27710, USA; satish.chitneni@duke.edu (S.K.C.); eftychia.koumarianou@abx-cro.com (E.K.); ganesan.v@duke.edu (G.V.); 2 ABX-CRO Advanced Pharmaceutical Services, D-01307 Dresden, Germany

**Keywords:** trastuzumab, HER2, radioiodination, SGMIB, SIB, breast cancer

## Abstract

Trastuzumab is an antibody used for the treatment of human epidermal growth factor receptor 2 (HER2)-overexpressing breast cancers. Since trastuzumab is an internalizing antibody, two factors could play an important role in achieving high uptake and prolonged retention of radioactivity in HER2-positive tumors after radioiodination—residualizing capacity after receptor-mediated internalization and susceptibility to dehalogenation. To evaluate the contribution of these two factors, trastuzumab was radiolabeled using the residualizing reagent *N*-succinimidyl 4-guanidinomethyl-3-[*I]iodobenzoate ([*I]SGMIB) and the nonresidualizing reagent *N*-succinimidyl-3-[*I]iodobenzoate ([*I]SIB), both of which are highly dehalogenation-resistant. Paired-label uptake and intracellular retention of [^125^I]SGMIB-trastuzumab and [^131^I]SIB-trastuzumab was compared on HER2-expressing BT474 human breast carcinoma cells. Tumor uptake and normal tissue distribution characteristics for the two labeled conjugates were assessed in mice bearing BT474M1 xenografts. The internalization and intracellular retention of initially-bound radioactivity in BT474 cells was similar for the two labeled conjugates up to 4 h, but were significantly higher for [^125^I]SGMIB-trastuzumab at 6 and 24 h. Similarly, [*I]SGMIB labeling resulted in significantly higher uptake and retention of radioactivity in BT474M1 xenografts at all studied time points. Moreover, tumor-to-tissue ratios for [^125^I]SGMIB-trastuzumab were consistently higher than those for [^131^I]SIB-trastuzumab starting at 12 h postinjection. Thus, optimal targeting of HER2-positive breast cancers with a radioiodinated trastuzumab conjugate requires an acylation agent that imparts residualizing capacity in addition to high stability towards dehalogenation in vivo.

## 1. Introduction

Human epidermal growth factor receptor 2 (HER2) is a transmembrane tyrosine kinase receptor that is overexpressed in about 22% of primary breast cancers and in multiple other cancers with varying frequency [1,2]. In breast cancers, HER2 overexpression is associated with aggressive tumor phenotype, resistance to chemotherapy and poor prognosis [3]. The significance of HER2 in breast cancer has led to the development of a number of HER2-targeted therapeutic agents including trastuzumab, a humanized monoclonal antibody (mAb) that binds to the extracellular domain of HER2 [4,5]. Two FDA-approved methods are currently used to determine HER2 status for trastuzumab therapy in breast cancers—immunohistochemical (IHC) analysis of HER2 protein expression and fluorescence in situ hybridization (FISH) analysis of HER2 gene amplification of tumor biopsy material [6]. Significant advances have been made toward developing radioimmunoconjugates to not only allow noninvasive imaging of HER2-overexpression in tumors by radionuclide-based imaging techniques, including PET or SPECT, but also for targeted radiotherapy. In this regard, trastuzumab has been labeled with a wide variety of radionuclides, including ^111^In, ^177^Lu, ^64^Cu, ^89^Zr, ^211^At, and radioiodine [7,8,9,10,11]. Preclinical and early clinical studies have demonstrated the promise of these agents for imaging HER2 overexpression in primary and metastatic breast cancer, and for imaging early tumor response to HER2-directed therapies [12,13,14]. Additionally, several smaller-size immunoconjugates, such as affibodies (MW: 6–7 kDa) and single-domain antibody-fragments (VHH molecules; MW: 12–15 kDa), are currently being evaluated for imaging and targeted radiotherapy of HER2-positive breast cancers [10,15,16]. As discussed below, the in vivo properties of these HER2-targeted agents depend on many factors with the choice of radionuclide and labeling method being critical parameters.

**Figure 1:**
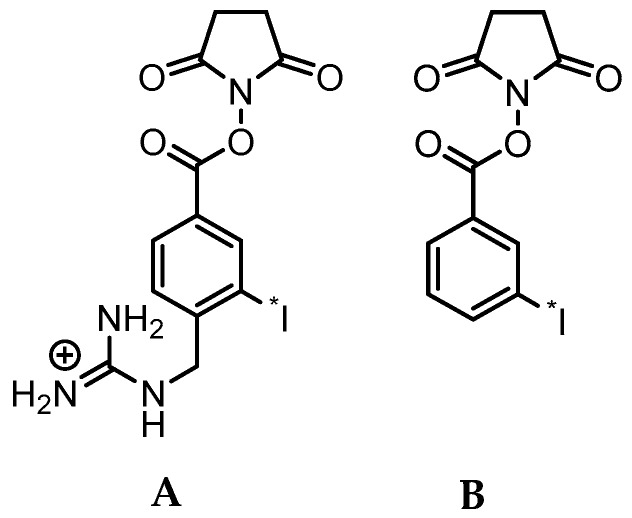
Chemical structures of the *N*-succinimidyl ester acylation agents [*I]SGMIB (**A**) and [*I]SIB (**B**).

In that regard, it is important to note that upon binding to HER2, trastuzumab undergoes internalization and presumably lysosomal degradation [17]. In general, this metabolic process can cause the loss of the radiolabel from the mAb and subsequent exportation of radiolabel and/or radiolabeled catabolites from tumor cells, potentially leading to a decrease in overall tumor radioactivity levels and tumor-to-background tissue ratios in vivo [18]. Standard methods for labeling mAbs with radiometals, such as ^111^In, ^177^Lu, ^64^Cu, and ^89^Zr, are generally thought to be residualizing or produce labeled catabolites that are trapped in tumor cells upon internalization, resulting in prolonged uptake of radioactivity in tumors [19,20]. On the other hand, the standard method for protein radioiodination (direct iodination, e.g., with Iodogen) is not suitable for labeling internalizing mAbs like trastuzumab for two reasons: susceptibility to dehalogenation and generation of low molecular weight radiocatabolites that can escape from targeted cells by passive transport [19,20]. With regard to the former, we and others developed acylation agents such as *N*-succinimidyl-3-[*I]iodobenzoate ([*I]SIB) (Figure 1) that contain a radioiodinated template that is resistant to deiodination in vivo as demonstrated by up to 100-fold decreased uptake of radioiodine in thyroid [21,22]. With regard to the later, several residualizing agents have been developed for the radioiodination of internalizing mAbs, which generate radiolabeled catabolites that are charged at lysosomal pH, thereby minimizing the escape of radioactivity from cancer cells after receptor-mediated internalization [23,24,25]. The most widely used of these reagents, *N*-succinimidyl 4-guanidinomethyl-3-[*I]iodobenzoate ([*I]SGMIB), is an analogue of [*I]SIB bearing a highly basic guanidino group at the 4-position (Figure 1).

Previous studies have shown that radioiodination of an epidermal growth factor receptor variant III (EGFRvIII)-targeted internalizing mAb with [*I]SGMIB provided a 3-4-fold increase in retention of radioactivity in target cells compared to that for the same mAb labeled via the Iodogen method [23]. Since [*I]SGMIB imparts a similarly low degree of deiodination when used to label proteins as [*I]SIB, it is not known the degree to which its low deiodination or its purported residualization are the dominant factor influencing the favorable results obtained when [*I]SGMIB is used to label internalizing mAbs. To address this issue, we have directly compared the HER2 targeting properties of trastuzumab labeled using [*I]SIB and [*I]SGMIB in HER2-positive BT474 breast carcinoma cells and BT474M1 tumor xenograft models. Our results demonstrate that the residualizing properties of [*I]SGMIB are a critical component in maximizing the tumor uptake and retention of radioactivity from radiolabeled internalizing mAbs, such as trastuzumab.

## 2. Results

### 2.1. Radiolabeling and Quality Control

Boc-protected [^125^I]SGMIB was obtained in 39 ± 8% yield, and Boc deprotection after high-performance liquid chromatography (HPLC) purification proceeded quantitatively to obtain [^125^I]SGMIB. [^131^I]SIB was synthesized with a radiochemical yield of 77 ± 15%. Conjugation yields for coupling [^125^I]SGMIB and [^131^I]SIB to trastuzumab were 45 ± 26% and 42 ± 10%, respectively. Radiochemical purity of the labeled conjugates determined by instant thin layer chromatography (iTLC) was >98%, as was protein-associated radioactivity assessed by the trichloroacetic acid (TCA) precipitation. The immunoreactive fractions for [^125^I]SGMIB-trastuzumab and [^131^I]SIB-trastuzumab were 71.3 ± 1.1% and 71.4 ± 0.9%, respectively.

### 2.2. Uptake, Internalization, and Cellular Processing on BT474 Cells In Vitro

**Figure 2:**
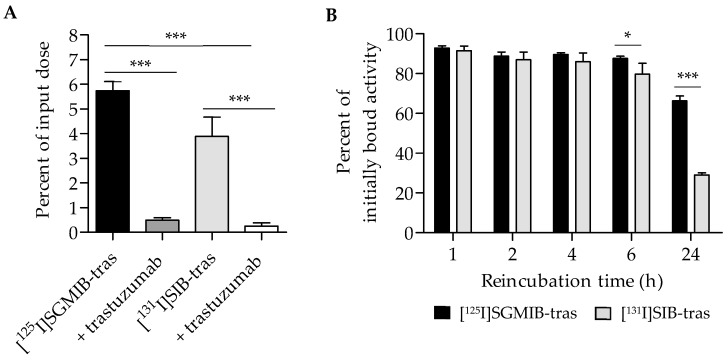
Paired-label studies of [^125^I]SGMIB-trastuzumab and [^131^I]SIB-trastuzumab in HER2-expressing BT474 cells in vitro. (**A**). Baseline uptake determined by incubating cells with the two labeled conjugates at 4 °C for 1 h. (**B**). Percentage of initially bound radioactivity retained by the cells after incubation of cells with fresh medium at 37 °C for various time points. * *p* < 0.05, *** *p* < 0.001.

The uptake and cellular processing of the two trastuzumab radioconjugates were compared in paired-label format on HER2-expressing BT474 human breast carcinoma cells. Figure 2A shows the uptake in cells during the initial incubation at 4 °C, selected to minimize internalization at this stage of the assay. After a 1-h incubation, 5.7 ± 0.9% of input radioactivity was cell-associated for [^125^I]SGMIB-trastuzumab compared to 3.9 ± 0.8% for [^131^I]SIB-trastuzumab, suggesting significantly higher uptake for trastuzumab labeled by the [*I]SGMIB method (*p* < 0.001). Addition of a 100-fold molar excess of unlabeled trastuzumab decreased cell uptake by >90%, indicating that the binding of the labeled trastuzumab conjugates to BT474 cells was HER2 specific (*p* < 0.001; Figure 2A). 

Total cell-associated, as well as intracellular, retention of radioactivity for the two trastuzumab conjugates were evaluated as a function of time. Figure 2B shows the percent of initially bound activity retained in cells (membrane-bound plus intracellular) after a 1–24 h incubation at 37 °C. No significant differences in total cell-associated activity between the two trastuzumab radioconjugates were observed up to 4 h with the mean percent of initially bound activity remaining cell-associated being >90% at 1 h and ~88% after 4 h. However, at 6 h, the cell-associated radioactivity for [^125^I]SGMIB-trastuzumab (87.6 ± 1.1%) was significantly higher than that for [^131^I]SIB-trastuzumab (79.8 ± 5.3%; *p* < 0.05). At 24 h, cell-associated radioactivity for [^131^I]SIB-trastuzumab decreased considerably, with only 29.1 ± 1.1% of initially-bound radioactivity remaining cell-associated compared to 66.3 ± 2.5% for [^125^I]SGMIB-trastuzumab (*p* < 0.001), representing a 2.3-fold retention advantage for the [^125^I]SGMIB conjugate.

**Figure 3:**
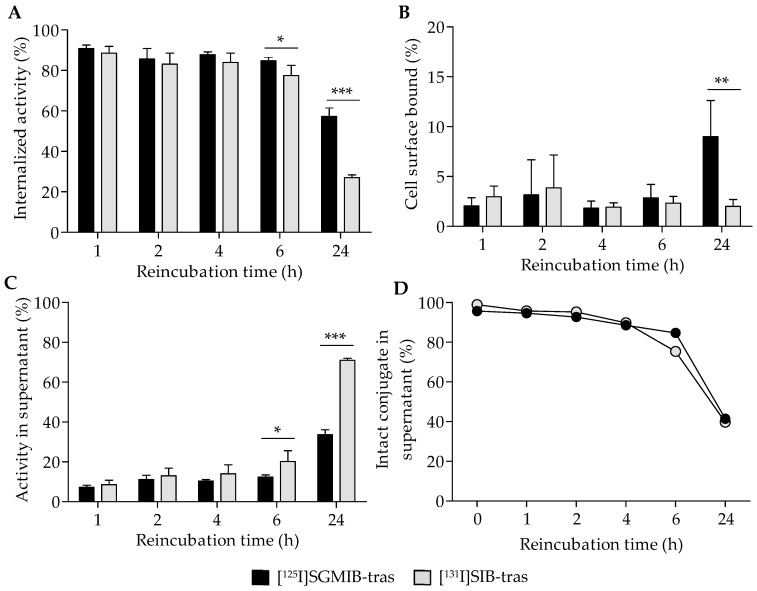
Distribution of initially bound radioactivity (shown in Figure 2B) in BT474 cells and the supernatant for [^125^I]SGMIB-trastuzumab and [^131^I]SIB-trastuzumab. Percent of the total cell-associated activity that had internalized into cells (**A**), bound to the cell surface (**B**), or released back into the supernatant (**C**) with time at physiologic conditions (37 °C). (**D**) Protein-associated activity in cell supernatants determined by the TCA precipitation assay. * *p* < 0.05, ** *p* < 0.01, *** *p* < 0.001.

Further analysis of the cell-associated radioactivity revealed that most of the radioactivity was internalized (Figure 3A) with a minor fraction found on the cell surface (Figure 3B). At 24 h, 57.3 ± 4.1% of initially-bound radioactivity remained intracellular for [^125^I]SGMIB-trastuzumab compared to only 27.1 ± 1.3% for [^131^I]SIB-trastuzumab (*p* < 0.001; Figure 3A). As expected, the cell culture supernatant activity profiles were complementary to their cell-associated radioactivity (Figure 3C). At 24 h, approximately 71% of the initially-bound radioactivity had leaked into the cell culture supernatant for [^131^I]SIB-trastuzumab, a level about twofold greater than that for [^125^I]SGMIB-trastuzumab (34%; *p* < 0.001). TCA precipitation analysis revealed nearly identical protein-associated activity in cell culture supernatants for both the labeled conjugates (Figure 3D), suggesting that higher cellular retention of radioactivity observed for [^125^I]SGMIB-trastuzumab at 24 h did not reflect differences in dissociation of intact labeled conjugates from the cells.

### 2.3. Tissue Distribution in Mice with BT474M1 Tumor Xenografts

A paired-label experiment was performed in NOD.SCID.gamma (NSG) mice bearing subcutaneous BT474M1 breast carcinoma xenografts to directly compare the tissue distribution of [^125^I]SGMIB-trastuzumab and [^131^I]SIB-trastuzumab. Uptake of [^125^I]SGMIB-trastuzumab in tumors was significantly higher than that for [^131^I]SIB-trastuzumab at all time points (*p* < 0.05), with the tumor retention advantage increasing with time (Table 1). With [^125^I]SGMIB-trastuzumab, tumor uptake increased to 20.3 ± 6.4% ID/g at 12 h, and remained nearly constant until the last studied time point (48 h; 20.1 ± 7.4% ID/g). In contrast, tumor uptake of [^131^I]SIB-trastuzumab peaked at 12 h (15.1 ± 3.7% ID/g) and decreased to 12.8 ± 4.2% ID/g at 48 h with the result that at 48 h, tumor uptake of [^125^I]SGMIB-trastuzumab was about 57% higher than that for the co-administered [^131^I]SIB-trastuzumab conjugate.

Unlike the behavior observed in tumors, the normal tissue distribution of [^125^I]SGMIB-trastuzumab and [^131^I]SIB-trastuzumab were, in general, quite similar with both radioconjugates clearing from most tissues with time (Table 1). Radioiodine activity in blood was slightly lower for [^125^I]SGMIB-trastuzumab compared to that for [^131^I]SIB-trastuzumab. For example, 18.0 ± 3.0% ID/g and 19.3 ± 3.0% ID/g was present in the blood at 12 h for [^125^I]SGMIB-trastuzumab and [^131^I]SIB-trastuzumab, decreasing to 7.0 ± 2.9% ID/g and 8.0 ± 3.1% ID/g at 48 h, respectively. Of note, thyroid uptake of ^131^I and ^125^I activity was not significantly different, and <0.3% ID beginning at 24 h, indicating similarly low deiodination of the two radioiodinated trastuzumab conjugates.

**Figure 4:**
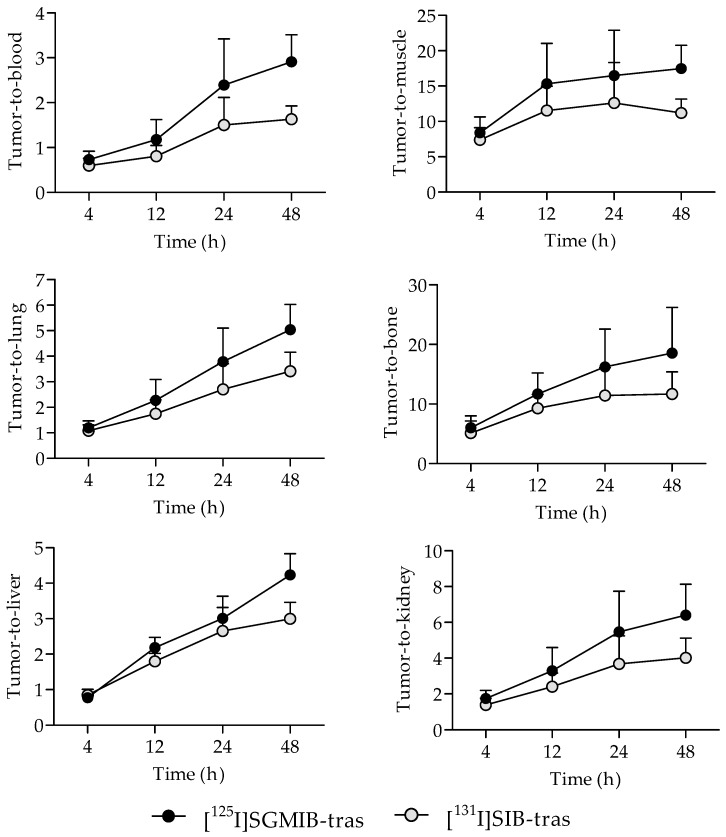
Comparison of the tumor-to-normal tissue ratios for [^125^I]SGMIB-trastuzumab and [^131^I]SIB-trastuzumab at 4–48 h after injection in NSG mice bearing subcutaneous BT474M1 xenografts.

Tumor-to-normal tissue ratios for [^125^I]SGMIB-trastuzumab were also higher compared to those for [^131^I]SIB-trastuzumab as early as 4 h after injection (Figure 4). Tumor-to-blood ratios for both radioiodinated trastuzumab conjugates were <1.0 at 4 h but increased with time, reaching 2.9 ± 0.6 for [^125^I]SGMIB-trastuzumab and 1.6 ± 0.3 for [^131^I]SIB-trastuzumab at 48 h (*p* < 0.001). Likewise, tumor-to-muscle ratios also increased with time, from 8.4 ± 2.2 and 7.4 ± 1.7 at 4 h, to 17.5 ± 3.3 and 11.2 ± 2.0 at 48 h for [^125^I]SGMIB-trastuzumab and [^131^I]SIB-trastuzumab, respectively. At 48 h, tumor-to-tissue ratios for [^125^I]SGMIB-trastuzumab in the liver, spleen, lungs, kidneys, and bone were 4.2 ± 1.4, 2.6 ± 0.7, 5.0 ± 1.0, 6.4 ± 1.7, and 18.5 ± 7.7, all values that were significantly higher (*p* < 0.05–0.001) than those observed for [^131^I]SIB-trastuzumab.

## 3. Discussion

A distinctive advantage of radioiodine for the development of theranostic agents for imaging and targeted radiotherapy of cancer is the availability of multiple radionuclides for imaging (e.g., ^123^I and ^131^I for SPECT, ^124^I for PET) and radiotherapy (^131^I β-particle, ^123^I and ^125^I, Auger electron emitters with an average Auger and Coster–Kronig electron energy released per decay of 7.4 keV and 12.2 keV, respectively [26]), thus providing multiple options. Furthermore, given the similarities in the chemical characteristics of iodine and the α-emitter ^211^At (t_1/2_: 7.2 h), both of which are halogens, radioiodinated mAbs could be developed as companion imaging agents for therapeutic radioimmunoconjugates labeled with ^211^At.

In this study, we have investigated the roles of residualization and dehalogenation on the performance of *N*-succinimidyl ester acylation agents for the radioiodination of internalizing mAbs such as trastuzumab using the previously validated acylation agents [*I]SGMIB and [*I]SIB. Although there is some disagreement about the extent and rate of its internalization, we have measured an internalization rate constant of (2.1 ± 0.3) × 10^−5^ s^−1^ on BT474 breast carcinoma cells [27], the cells used in the current study. Both [*I]SGMIB and [*I]SIB contain the dehalogenation resistant 3-[*I]iodophenyl moiety [22,28], allowing direct comparison of the effectiveness of the residualizing guanidino group on tumor uptake; likewise, it permits direct comparison of the potential effects of the guanidino group on dehalogenation as well as other aspects of normal tissue radioactivity distribution.

From a radiochemistry perspective, [*I]SIB offers significant advantages for mAb radioiodination including a shorter synthesis time ([*I]SIB, 90 min; [*I]SGMIB, 140 min) and higher radiochemical yield ([*I]SIB, 77 ± 15%; [*I]SGMIB, 39 ± 8%), consistent with previous reports [28,29]. On the other hand, the results from the present study demonstrate improved tumor retention both in vitro and in vivo when trastuzumab was labeled using [*I]SGMIB, confirming that the residualizing labeling approach was the better reagent for labeling this internalizing mAb. This likely reflects the higher intracellular retention of small-molecule labeled catabolities from [*I]SGMIB-trastuzumab after HER2-mediated internalization and cellular processing of the labeled conjugate. Indeed, the levels of radioactivity for [*I]SGMIB-trastuzumab retained in BT474 cells in the present study were significantly higher (about two-fold; 57.3 ± 4.1%) than for [*I]SIB-trastuzumab (27.1 ± 0.7%) at 24 h.

Previous studies have evaluated the nature of the small molecule labeled catabolites generated from mAbs and mAb fragments labeled with these reagents. With [*I]SIB, these were primarily the lysine conjugate of iodobenzoic acid (IBA-Lys), and to a lesser extent, the glycine conjugate of iodobenzoic acid (IBA-Gly) as well as free iodobenzoic acid [20]. When [*I]SGMIB was used to label an internalizing mAb, the labeled catabolites were 4-guanidinomethyl-3-iodobenzoic acid (GMIBA; minor) and its glycine conjugate (GMIBA-Gly; major) [23]. It was unexpected that GMIBA-Lys was not observed, which was attributed to possible proteolytic cleavage of the GMIBA-Lys bond followed by the reaction of the cleaved GMIBA with glycine to form GMIBA-Gly inside the cells [20,23]. Nonetheless, GMIBA-Gly is expected to remain trapped in cells because of the presence of the highly basic guanidino function (p*K*_a_ ~13) in GMIBA [23]. Thus, the labeled catabolites of [*I]SGMIB-trastuzumab are expected to be not only more hydrophilic than those generated from [*I]SIB-labeled mAb, but also remain protonated at the lysosomal pH (~4.5–5.0), thereby limiting their ability to escape tumor cells by passive diffusion. Consistent with these properties, a significantly higher percentage of radioactivity in cell culture supernatant was not associated with protein with [^131^I]SIB-trastuzumab in the current study, reflecting the release of a higher level of low-molecular weight radiocatabolites with this reagent (Figure 3C,D). As noted in Table 1, the superior intracellular retention observed with [^125^I]SGMIB-trastuzumab translated into higher and more prolonged tumor uptake compared to mAb labeled using the non-residualizing [*I]SIB reagent.

The potential advantages of residualizing labeling for enhancing tumor retention could be of limited theranostic advantage if the approach also results in increased uptake in normal tissues, a constraint that should be considered with both radiohalogens and radiometals. With regard to the former, labeling trastuzumab with [*I]SGMIB not only increased radioactivity retention in BT474M1 xenografts but also improved tumor-to-normal tissue ratios including in the kidney and the liver compared with mAb labeled using [*I]SIB. In contrast, previous studies labeling trastuzumab with two D-peptide based residualizing agents showed higher uptake of radioiodine than we observed for [^125^I]SGMIB-trastuzumab in the same xenograft model; however, kidney retention with these D-peptide agents was considerably higher and more persistent [24,25], making [*I]SGMIB the reagent of choice for trastuzumab radioiodination.

Due to the differences in xenograft models and other variables, such as protein dose, comparison of biodistribution results described in the literature with radiolabeled trastuzumab conjugates must be done with caution. With regard to studies evaluating trastuzumab labeled with radiometals, these approaches generally are considered to be residualizing and have shown superior tumor localization compared with trastuzumab labeled using the non-residualizing Iodogen method [30]. On the other hand, the uptake of [*I]SGMIB-trastuzumab in BT474M1 tumors in the present study compare favorably with those reported for ^89^Zr- and ^64^Cu-labeled trastuzumab conjugates, which exhibited ~14–30% ID/g in tumor at 24–96 h post injection [8,31,32,33,34]. A notable exception is the study by Holland et al. wherein 45.1 ± 7.6% ID/g tumor uptake was observed for ^89^Zr-DFO-trastuzumab in athymic mice bearing BT474 xenografts [14], significantly higher than the values observed for [*I]SGMIB-trastuzumab in the BT474M1 model.

As noted above, the residualizing nature of ^64^Cu- and ^89^Zr-labeled mAbs and/or the catabolites generated from them is advantageous with regard to maximizing tumor retention of radioactivity; however, there also can be an intrinsic disadvantage to radiometal labeling of proteins. A number of preclinical and clinical studies have shown that ^64^Cu- and ^89^Zr-labeled mAb conjugates exhibit high uptake of the radiometal in the liver and/or the bone [9,33,35,36]. Since liver and bone are among the most common sites of metastasis for breast cancers, this normal tissue uptake pattern can potentially compromise the sensitivity of ^64^Cu- and ^89^Zr-labeled trastuzumab conjugates for detecting metastatic lesions in these sites [18,37,38]. In a recent study evaluating the usefulness of ^89^Zr-labeled trastuzumab for detecting tumor lesions in breast cancer patients, Dehdashti et al. found that ^89^Zr-trastuzumab was unable to discriminate effectively between HER2-positive and HER2-negative lesions in the liver; moreover, lesion uptake was similar to radioactivity levels observed in the normal liver [39]. Similar discordance between ^89^Zr-trastuzumab uptake in PET imaging and HER2 status (determined by IHC) as well as a high degree of false-positive suspicious ^89^Zr-avid foci also has been reported in other studies [12,40]. These observations point to the need for continued development and optimization of HER2 targeted radioimmunoconjugates to identify an agent with low accumulation of radioactivity in liver, bone and other frequently occurring metastatic sites. 

A recent study evaluated the effect of reducing binding to Fc-γ-receptors on immune cells performed with ^89^Zr-DFO-trastuzumab in NSG mice with subcutaneous BT474 xenografts [34] provides an excellent framework for comparison to the results obtained in the current study in a nearly identical xenograft model. With ^89^Zr-DFO-trastuzumab, uptake in BT474 xenografts at 24 h (27.6 ± 10.7% ID/g vs. 20.7 ± 7.0% ID/g for [^125^I]SGMIB) and 48 h (17.1 ± 2.4% ID/g vs. 20.1 ± 7.4% ID/g for [^125^I]SGMIB) was quite similar to the results in the current study for [^125^I]SGMIB-trastuzumab; however, tumor-to-liver (^89^Zr, 1.0 ± 0.2; ^125^I, 4.2 ± 1.4 at 48 h) and tumor-to-bone (^89^Zr, 1.3 ± 0.3; ^125^I, 18.5 ± 7.7 at 48 h) ratios were considerably lower for ^89^Zr-DFO-trastuzumab. Hypothesizing that the high liver (and spleen) uptake was caused by binding of the Fc region of the mAb to Fc-γ-receptors on immune cells, the authors then developed deglycosylated versions of trastuzumab which, when labeled by ^89^Zr-DFO conjugation, exhibited up to a 3.5-fold decrease in liver activity compared with the original ^89^Zr-DFO-trastuzumab. Moreover, a concomitant increase in tumor uptake was observed with a tumor uptake of 76.8 ± 10.1% ID/g at 48 h seen for the ^89^Zr-^nss^trastuzumab-PNGaseF construct in the same BT474 xenograft NSG model. In summary, this is a very promising strategy whose benefits could be radionuclide and labeling method agnostic, and in future studies we shall evaluate its potential utility in combination with [*I]SGMIB labeling of trastuzumab.

## 4. Materials and Methods

### 4.1. General

Chemicals and solvents were purchased from VWR International (Suwanee, GA, USA) or Sigma-Aldrich (St. Louis, MO, USA), and were used as supplied. Radioiodine was obtained from PerkinElmer (Waltham, MA, USA) as sodium [^125^I]iodide and sodium [^131^I]iodide in 0.1 M NaOH solution, with a molar activity of about 80 GBq/mmol and 70 GBq/mmol, respectively. Prior to labeling, trastuzumab (Herceptin^®^, Genentech, Inc., South San Francisco, CA, USA) was buffer-exchanged into 0.1 M borate buffer, pH 8.5 using a 30-kDa molecular weight cut-off concentrator (Vivaspin^®^ 2, GE Healthcare Life Sciences, Piscataway, NJ, USA). Purification of the radioiodinated prosthetic agents *N*-succinimidyl 3-[^131^I]iodobenzoate ([^131^I]SIB) and *N*-succinimidyl 4-guanidinomethyl 3-[^125^I]iodobenzoate ([^125^I]SGMIB) was performed on a Beckman Gold^®^ HPLC system that was equipped with a gradient solvent module and a variable wavelength UV detector (Beckman Coulter Inc., Brea, CA, USA). The effluent was passed through a LabLogic radio-HPLC detector (LabLogic; Brandon, FL, USA) for detection of the radiometric signal. Data were acquired and analyzed using the software (32 Karat^®^) provided by the vendor (Beckman Coulter, Inc.). 

### 4.2. Synthesis of N-Succinimidyl 3-[^131^I]iodobenzoate ([^131^I]SIB)

Synthesis and purification of [^131^I]SIB was performed as reported previously [29]. Briefly, ^131^I (148–185 MBq) in 2–6 µL of 0.1 M NaOH was dispensed into a half-dram vial and the radioactivity was concentrated using a gentle stream of argon. To the vial were added 3 µL of acetic acid (3% in chloroform; *v*/*v*) and 5 µL of *tert*-butyl hydroperoxide (TBHP; 10% in chloroform; *w*/*v*) followed by the tin precursor, *N*-succinimidyl 3-(tri-*n*-butylstannyl)benzoate (0.1 mg in 10 µL chloroform). The labeling reaction mixture was then incubated at room temperature (RT) for 15–20 min, and the crude mixture was purified by normal-phase HPLC using an Alltech Partisil^™^ silica column (10 µm; 250 × 4.6 mm) eluted in isocratic mode with 25% ethyl acetate in hexanes containing 0.2% acetic acid as described [27]. The solvents were evaporated using argon and the dried [^131^I]SIB was used for conjugation with trastuzumab.

### 4.3. Synthesis of N-Succinimidyl 4-guanidinomethyl 3-[^125^I]iodobenzoate ([^125^I]SGMIB)

Synthesis of [^125^I]SGMIB was performed following a procedure similar to that described above for [^131^I]SIB but using sodium [^125^I]iodide (74–222 MBq) and the corresponding Boc-protected tin precursor *N*-succinimidyl 4-[*N*^1^,*N*^2^-bis(*tert*-butyloxycarbonyl)guanidinomethyl]-3-(trimethyl-stannyl)benzoate (Boc_2_-SGMTB; 0.05 mg) [23]. The labeled intermediate Boc_2_-[^125^I]SGMIB was purified by normal-phase HPLC (*t*_R_ = 23 min) and the pooled HPLC fractions containing Boc_2_-[^125^I]SGMIB was evaporated/dried as described for [^131^I]SIB. Next, Boc_2_-[^125^I]SGMIB was treated with trifluroacetic acid (TFA; 100 µL) at RT to remove the Boc groups as described [23]. The dried [^125^I]SGMIB was then used for labeling trastuzumab as described below.

### 4.4. Conjugation of [^125^I]SGMIB and [^131^I]SIB to Trastuzumab 

To the dried [^125^I]SGMIB (22–63 MBq) or [^131^I]SIB (46–149 MBq) was added trastuzumab in 0.1 M borate buffer, pH 8.5 (0.1 mg in 50–77 µL) and the mixture was incubated at RT for 20 min and then purified using a PD–10 desalting column (GE Healthcare Life Sciences) eluted with PBS, pH 7.4. The protein-associated radioactivity was determined by instant thin layer chromatography (iTLC) using PBS at pH 7.4 as the mobile phase, and by co-precipitation with human serum albumin (5%, Grifols Biologicals Inc., Los Angeles, CA, USA) using trichloroacetic acid (TCA, 20%) [25]. Immunoreactivity was determined by the Lindmo method using magnetic beads coated with HER2 extracellular domain as described previously [25].

### 4.5. Uptake and Internalization of Labeled Trastuzumab Conjugates in HER2-Expressing BT474 Cells

The uptake and intracellular retention of radioactivity from the [^125^I]SGMIB-trastuzumab and [^131^I]SIB-trastuzumab were evaluated on HER2-expressing BT474 human breast carcinoma cells in paired-label format. Approximately 24 h before the uptake experiments, cells were plated in six-well plates at a density of 8 × 10^5^ cells/well in DMEM/F12 medium (2 mL) supplemented with fetal bovine serum (FBS, 10%), streptomycin (100 μg/mL), and penicillin (100 IU/mL). On the day of the experiment, plates were incubated at 4 °C for 0.5 h, incubation medium was removed, and cells were rinsed with cold PBS (3 × 1 mL). Aliquots of cold media (fresh, 1.8 mL) were then added to the cells followed by addition of [^131^I]SIB-trastuzumab and [^125^I]SGMIB-trastuzumab (1.5 µg/0.1 mL of each). Cells were incubated at 4 °C for 1 h, incubation medium was removed, cells rinsed with cold PBS (2 × 1 mL) and incubated at 37 °C with fresh medium for 1, 2, 4, 6, or 24 h (*n* = 3 at each time point). To determine nonspecific binding, cells were evaluated in parallel wells with the labeled conjugates in the presence of a 100-fold excess of unlabeled trastuzumab (20 µL; 0.3 mg protein). The unbound radioactivity, cell surface-bound radioactivity, and the internalized radioactivity were determined as described [25]. Aliquots of each supernatant were mixed with 10% HSA (0.25 mL) and 20% TCA (0.15 mL) to determine the percentage of radioactivity in the cell culture media that was protein-associated. Data are presented as a mean for three wells with an average value determined from two independent experiments.

### 4.6. Evaluation of the Labeled Trastuzumab Conjugates in BT474M1 Tumor Xenografts In Vivo 

The tissue distribution of trastuzumab labeled with [^125^I]SGMIB and [^131^I]SIB was evaluated in NOD.SCID.gamma (NSG) mice bearing subcutaneous HER2-expressing BT474M1 human breast carcinoma xenografts. The animal experiments were conducted in accordance with the guidelines of Duke University’s Institutional Animal Care and Use Committee (IACUC) and following an approved IACUC protocol (#A246-18-10). Tumor xenografts were generated using BT474M1 cells, a more tumorigenic version of the parental BT474 human breast carcinoma cell line, as described previously [15]. Tissue distribution study was initiated when tumors reached a volume of 250–500 mm^3^. For this, groups of five animals were injected with a mixture of [^125^I]SGMIB-trastuzumab and [^131^I]SIB-trastuzumab (37 kBq/1.5 µg each; 100 µL PBS) via the tail vein, and the animals were euthanized by isoflurane overdose at 4 h, 12 h, 24 h, or 48 h post injection. Tissues of interest were collected, weighed and counted for radioactivity in an automated gamma counter. The results were calculated as % injected dose (% ID) or % injected dose per gram (% ID/g) and tumor-to-tissue ratios. 

### 4.7. Statistical Analysis 

Unless otherwise stated, cell and tissue uptake data are presented as mean ± standard deviation. Statistical analyses of the data were performed by the paired two-tailed Student’s *t*-test, and differences at the 95% confidence level (*p* < 0.05) were considered to be statistically significant.

## 5. Conclusions

Radioiodination of trastuzumab using the residualizing agent [*I]SGMIB provides significantly higher residualizing capacity (by ~60%) compared to that for the non-residualizing but likewise dehalogenation-resistant labeling agent [*I]SIB. Thus, optimal targeting of HER2-positive breast cancers with a radioiodinated trastuzumab conjugate requires an acylation agent that is both residualizing and resistant to dehalogenation, to achieve high radioactivity concentrations in tumors. These results also support the evaluation of radiolabeled [*I]SGMIB-trastuzumab conjugates for imaging and/or treatment of patients with HER2-positive malignancies.

## Figures and Tables

**Table 1:** Paired-label biodistribution data for the [^125^I]SGMIB-trastuzumab (SGMIB) and [^131^I]SIB-trastuzumab (SIB) in NSG mice bearing subcutaneous BT474M1 xenografts, and expressed as % injected dose per gram tissue (% ID/g).

Organ/Tissue	4 h ^1^		12 h ^1^		24 h ^1^		48 h ^1^	
	SGMIB	SIB	SGMIB	SIB	SGMIB	SIB	SGMIB	SIB
Liver	17.2 ± 2.0	14.5 ± 2.9	9.3 ± 1.6	8.6 ± 1.4	8.2 ± 3.8	7.2 ± 4.0	5.0 ± 2.4	4.7 ± 2.4
Spleen	19.8 ± 9.0	19.1 ± 9.1	14.0 ± 8.2	12.8 ± 7.5	7.7 ± 3.1	7.0 ± 3.1	8.9 ± 6.4	8.3 ± 6.1
Lungs	11.1 ± 2.0	10.8 ± 2.0	9.1 ± 1.5	9.0 ± 1.7	5.8 ± 1.6	5.8 ± 1.7	4.1 ± 1.7	3.9 ± 1.6
Heart	6.9 ± 1.8	7.1 ± 2.0	5.7 ± 1.2	5.9 ± 1.3	3.4 ± 1.6	3.5 ± 1.6	2.5 ± 1.2	2.6 ± 1.3
Kidneys	7.6 ± 1.2	8.5 ± 0.9	6.5 ± 1.1	6.5 ± 1.0	4.1 ± 1.4	4.4 ± 1.4	3.3 ± 1.5	3.4 ± 1.5
Bladder	2.0 ± 0.6	2.2 ± 0.7	3.6 ± 0.6	3.6 ± 0.5	4.0 ± 2.1	4.2 ± 2.6	3.6 ± 1.4	3.6 ± 1.3
Stomach	2.7 ± 0.7	3.0 ± 0.7	1.8 ± 0.4	1.8 ± 0.5	1.7 ± 0.5	2.1 ± 0.6	1.1 ± 0.9	1.3 ± 1.0
Thyroid ^2^	0.6 ± 0.4	0.6 ± 0.4	0.8 ± 0.2	0.8 ± 0.2	0.3 ± 0.1	0.3 ± 0.1	0.2 ± 0.2	0.3 ± 0.2
Bone	2.3 ± 0.4	2.4 ± 0.4	1.7 ± 0.3	1.7 ± 0.5	1.4 ± 0.4	1.4 ± 0.4	1.2 ± 0.7	1.2 ± 0.7
Muscle	1.6 ± 0.3	1.6 ± 0.3	1.4 ± 0.2	1.4 ± 0.2	1.4 ± 0.5	1.3 ± 0.5	1.1 ± 0.3	1.1 ± 0.3
Blood	18.4 ± 4.5	19.9 ± 4.6	18.0 ± 3.0	19.3 ± 3.0	9.6 ± 3.6	10.6 ± 3.5	7.0 ± 2.9	8.0 ± 3.1
Brain	0.7 ± 0.1	0.8 ± 0.1	0.8 ± 0.2	0.8 ± 0.2	0.4 ± 0.1	0.4 ± 0.1	0.3 ± 0.1	0.3 ± 0.1
Tumor ^3^	13.0 ± 2.3	11.5 ± 2.3	20.3 ± 6.4	15.2 ± 3.7	20.7 ± 7.0	14.6 ± 4.3	20.1 ± 7.4	12.8 ± 4.2

^1^ % ID/g values; ^2^ % injected dose; ^3^ Difference in uptake is statistically significant between the two labeled conjugates for all time points.
